# Nocardiose pulmonaire sur un terrain immunocompétent: à propos de 2 cas

**DOI:** 10.11604/pamj.2017.27.149.12862

**Published:** 2017-06-29

**Authors:** Yasmina Rhofir, Rachida Zahraoui, Nabil Tiress, Hicham Naji-Amrani, Mouna Soualhi, Jamal Eddine Bourkadi

**Affiliations:** 1 Service de Pneumo-Phtisiologie, Hôpital Moulay Youssef, Rabat, Maroc; 2 Service de Pneumo-Phtisiologie, Hôpital Militaire d'Instruction Mohamed V, Rabat, Maroc

**Keywords:** Abcès sous cutané, immunocompétent, nocardia, nocardiose pulmonaire, Subcutaneous abscess, immunocompetent, nocardia, pulmonary nocardiosis

## Abstract

La nocardiose est une infection rare, mais sévère, causée par des bactéries du genre nocardia, qui appartiennent à l'ordre des actinomycétales. Si elles peuvent toucher l'adulte immunocompétent, les nocardioses restent des pathologies de l'individu fragilisé sur le plan immunitaire. L'atteinte pulmonaire reste la plus fréquente, sa prise en charge correcte est liée au diagnostic qui est souvent retardé par des présentations non spécifiques et des prélèvements non concluants. Nous rapportons ici deux cas de nocardiose chez des patients immunocompétents. Le premier cas est celui d'un homme de 24 ans, avec notion de tabagisme et d'éthylisme, hospitalisé pour des douleurs thoraciques et des hémoptysies de faible abondance, évoluant depuis deux mois, avec apparition d'abcès sous cutanés dorsaux fistulisés. L'exploration radiologique découvre une masse tissulaire médiastino-pulmonaire droite avec lyse costale adjacente et diffusion aux tissus para vertébraux droits. Les prélèvements bactériologiques restent négatifs motivant une biopsie scannoguidée de la lésion qui est revenue en faveur d'infection à nocardiose. Le second cas concerne un homme de 22 ans, aux antécédents de tuberculose pleurale traitée il y a 8 ans puis une rechute de tuberculose en 2011 (abcès médiastinal). Admis pour suspicion de rechute de tuberculose devant une toux chronique avec altération de l'état général et une hépatosplénomégalie. Le scanner thoracique montre des condensations alvéolaires avec pleurésie. Au cours de son hospitalisation, apparition de tuméfactions sous cutanées purulentes dont l'étude bactériologique du pus est revenue en faveur de nocardiose avec une souche résistante à tous les antibiotiques sauf colistine et bactrim. Les auteurs illustrent à travers ces deux observations, les aspects cliniques et radiologiques de nocardiose pulmonaire en mettant le point sur les difficultés diagnostiques et thérapeutiques surtout dans un pays à forte prévalence de tuberculose et très faible incidence de nocardiose.

## Abstract (en)

Nocardiosis is a rare but severe infection caused by bacteria of the genus nocardia, which belong to the order actinomycetales. If they can affect immunocompetent adult, nocardioses are pathologies affecting the individuals with weakened immune system. Pulmonary involvement is the most common manifestation, its correct management is based on diagnosis, which is often delayed due to non-specific symptoms and inconclusive specimens. We here report two cases of nocardiosis in immunocompetent patients. The first case concerns a 24-year old man with a history of smoking and alcoholism, hospitalized for chest pain and hemoptysis of low abundance evolving for two months, associated with the occurrence of dorsal subcutaneous fistulized abscess. Radiological assessment showed right mediastino-pulmonary tissue mass associated with adjacent costal lysis and dissemination in rights paravertebral tissues. Bacteriological sampling remained negative motivating ultrasound-guided biopsy of the lesion, which confirmed the diagnosis of nocardia infection. The second case concerns a 22-year old man with a history of pleural tuberculosis treated 8 years ago and of relapse of tuberculosis in 2011 (mediastinal abscess). He was admitted to hospital due to suspicion of relapse of tuberculosis based on chronic cough with alteration of general state and hepatosplenomegaly. Chest CT scan showed alveolar condensations with pleurisy. During his hospitalization, purulent subcutaneous swellings occurred. Bacteriological analysis of the pus confirmed the diagnosis of nocardiosis. Nocardia strains were resistant to all antibiotics except for colistin and bactrim. This study aims to highlight the clinical and radiological aspects of pulmonary nocardiosis, focusing on diagnostic and therapeutic difficulties especially in a country with a high prevalence of tuberculosis and a very low incidence of nocardiosis.

## Introduction

La nocardiose est une infection rare due à des bactéries filamenteuses, aérobies, présentes dans l'environnement, elle touche souvent des hôtes immunodéprimés, néanmoins, elle peut survenir chez des patients sans facteurs favorisants identifiables [1]. La localisation peut être unique ou multiple avec prédilection de l'atteinte pulmonaire.

## Patient et observation

### Observation n°1

**Figure 1:**
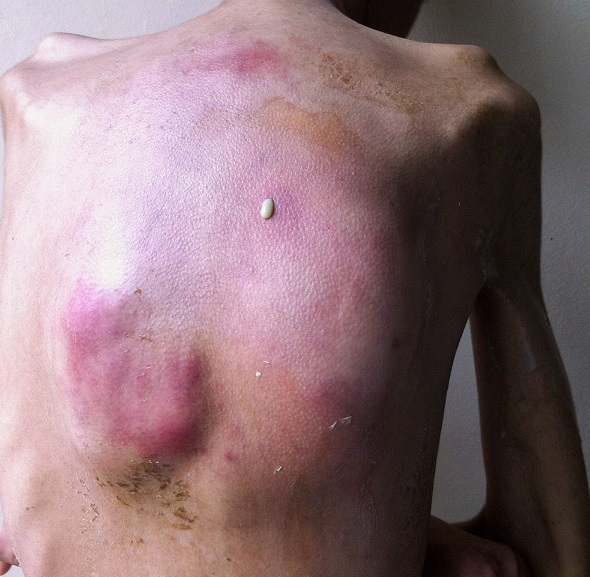
Abcès dorsal fistulisé

**Figure 2:**
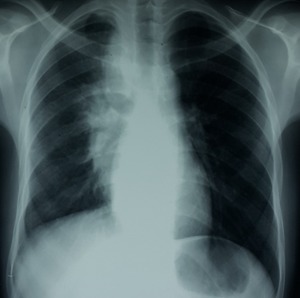
Radiographie thoracique de face montrant une opacité hétérogène suspecte médiastino-pulmonaire droite étendue

**Figure 3:**
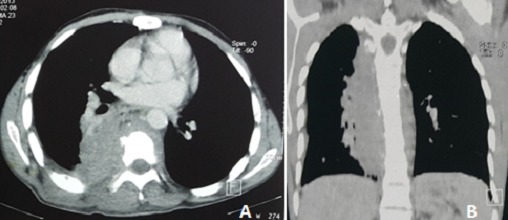
(A, B) tomodensitométrie thoracique en fenêtre médiastinale objectivant une lésion tissulaire para-vertébrale droite avec envahissement des parties molles

**Figure 4:**
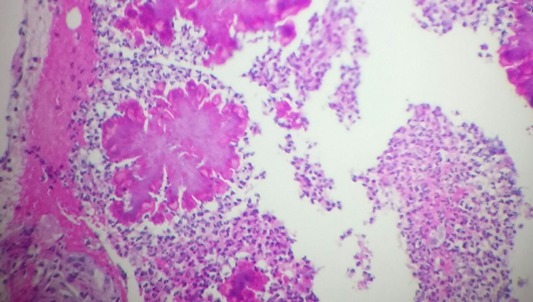
Lésion granulomateuse à centre abcédé avec couronne de polynucléaires neutrophiles (HE x 10)

**Figure 5:**
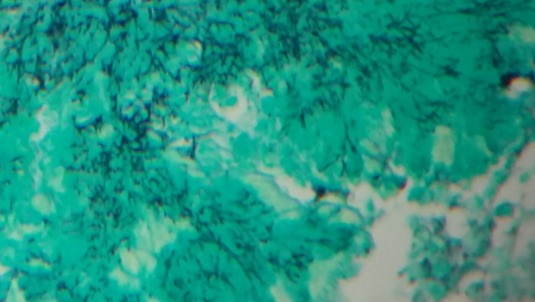
Bacille filamenteux mis en évidence par la coloration de Grocott (Grocott x 20)

Il s'agit d'un patient de 24 ans, ayant dans ses antécédents un tabagisme et alcoolisme chronique. Il est hospitalisé pour des douleurs thoraciques postérieures insomniantes avec une dyspnée stade 2 de la mMRC et crachats hémoptoïques évoluant depuis deux mois dans un contexte d'apyrexie et d'altération de l'état général. L'examen clinique trouvait un patient cachectique, ayant trois tuméfactions sous cutanés dorsales de consistance liquidienne douloureuse à la palpation dont la plus volumineuse mesure 15 cm de grand diamètre, associées à des signes inflammatoires en regard (Figure 1). L'examen pleuropulmonaire était normal, les aires ganglionnaires superficielles étaient libres. La radiographie et le scanner thoraciques (Figure 2, Figure 3) montraient une lésion médiastino-pulmonaire droite de densité tissulaire des lobes supérieurs et inférieurs avec lyse costale adjacente au niveau dorsal et diffusion aux tissus para vertébraux droits, ainsi que de multiples adénopathies médiastinales. L'examen direct (ED) dans les expectorations à la recherche de mycobactérium tuberculosis (BK) était négatif. Le bilan biologique objectivait un syndrome inflammatoire manifeste avec une proteine réactive (CRP) à 236 mg/l et une vitesse de sédimentation (VS) à 109 mm la première heure; une bicytopénie faite d'une anémie à 6.6 g/dl, hypochrome et microcytaire avec une thrombopénie à 113700/mm^3^motivant la réalisation d'une biopsie ostéomédulaire qui est revenue normale, évoquant un syndrome d'activation macrophagique; la fonction rénale, le bilan hépatique et la sérologie du virus humain de l'immunodéficience (HIV) étaient normaux. La fibroscopie bronchique était normale avec la recherche de germes et de BK dans le liquide du lavage bronchiolo-alvéolaire qui était négative à l'ED. L'évolution était marquée par la fistulisation des tuméfactions sous cutanées dorsales avec issue de pus, dont l'étude cytobactériologique a montré une nette prédominance de polynucléaires neutrophiles (PNN) avec absence de germes ou de BK à l'ED et à la culture. Une biopsie scannoguidée de la masse pulmonaire ayant montré à l'étude anatomopathologique une réaction granulomateuse à centre abcédé avec couronne de PNN (Figure 4) suivie d'une coloration de Grocott qui a mis en évidence des bacilles filamenteux caractéristiques du nocardia (Figure 5). Le patient a bénéficié d'une transfusion de culots globulaires, de soins locaux avec évacuation du pus et d'antibiothérapie à base d'amoxicilline protégée (3 g/j). Après trois semaines, l'évolution était favorable sur le plan clinique, biologique et radiologique avec décision de prolonger l'antibiothérapie pendant six mois.

### Observation n°2

Il s'agit d'un patient âgé de 22 ans, traité pour tuberculose pleurale en 2008 puis médiastinale en 2011. Il est hospitalisé pour suspicion d'une deuxième rechute de tuberculose devant la symptomatologie faite de toux chronique évoluant dans un contexte fébrile et d'altération de l'état général. L'examen clinique trouvait une hépato-splénomégalie, avec apparition au cours de son hospitalisation de plusieurs tuméfactions sous cutanées purulentes axillaires droites et dorsales et d'une paraplégie. La radiographie thoracique montrait des opacités hilaires bilatérales. Le bilan à la recherche de tuberculose était négatif (BK dans les expectorations et dans l'aspiration bronchique à l'ED et à la culture, ainsi que le Genexpert dans les expectorations). Le scanner thoracique a montré des condensations parenchymateuses pulmonaires avec extension vertébrale importante. Le bilan biologique objectivait un syndrome inflammatoire (CRP à 120 mg/l et VS à 80 mm). La sérologie HIV était négative. L'évolution était marquée par la fistulisation des tuméfactions et l'émission de pus dont l'étude bactériologique a mis en évidence à l'ED la présence de Nocardia sp. Le diagnostic de nocardiose diffuse a été retenu. L'antibiogramme avait montré une résistance aux céphalosporines et aminosides et une sensibilité à la colistine et au triméthoprim-sulfaméthoxazol. Une antibiothérapie a été démarrée en fonction de l'antibiogramme pendant 1 mois. Malheureusement, le patient est décédé dans un tableau de trouble de conscience suggérant une localisation cérébrale.

## Discussion

La nocardiose est une maladie rare, les séries comportent un petit nombre de patients de 2 à 7 sur plusieurs années [2]. Au Maroc, il s'agit du quatrième cas décrit de nocardiose: 1 cas d'abcès cérébral à nocardia [3], un autre cas de nocardiose pulmonaire et sarcoidose [4] et un troixième cas de pleurésie purulente à nocardia [5]. Les bactéries du genre nocardia, qui appartiennent à l'ordre des actinomycétales, sont des bactéries filamenteuses ramifiées ou pléiomorphes, à métabolisme aérobie strict. Ce sont des bactéries hydrotelluriques largement distribuées dans l'environnement, vivant à l'état saprophyte dans le sol et dont de nombreuses espèces sont pathogènes pour l'homme et l'animal ainsi que pour les plantes. Les principales espèces responsables de nocardiose pulmonaire: N. asteroides, N. farcinica, N. nova, N. otitidiscaviarum et N. transvalensis, ainsi que de nouvelles espèces, telles que N. cyriacigeorgica, N. abscessus, N. veterana et N. ignorata [1]. La fréquence de l'atteinte pleuro-pulmonaire varie de 44.3% à 85%, expliquée par le mode de contamination le plus fréquent: inhalation de fragments de filaments ou de spores présents dans l'air en particulier dans la poussière [2]. Les nocardioses restent des pathologies de sujets fragiles sur le plan immunitaire, notamment les sujets HIV positifs, la corticothérapie au long court, les transplantés, les porteurs de néoplasie solide ou hémopathie maligne, les atteintes hépatiques et rénales [6] ainsi que les pathologies cardiaques et anémie hémolytiques auto immunes [7]. Des facteurs débilitants ont aussi été décrits: tuberculose, broncho-pneumopathie chronique obstructive, asthme, dilatation de bronches ou pneumoconiose, sarcoïdose, alcoolisme chronique, diabète et malnutrition [1]. Néanmoins, certains cas ont été décrits chez des sujets sains et immunocompétents; dans la série de Kurahara, 88% des patients avaient une maladie sous-jacente et un seul cas était sous corticothérapie. Il est donc évident que la nocardiose pulmonaire peut se développer également chez des patients non immunodéprimés, mais souvent avec un terrain de débilité [6]. Les manifestations cliniques sont souvent non spécifiques et polymorphes. La présentation clinique la plus fréquente est celle d'une pneumonie subaiguë ou chronique souvent nécrosante [1]. La complication principale de cette contamination pulmonaire est la dissémination secondaire par voie hématogène, responsable de nocardiose systémique. Le site le plus fréquemment atteint dans ce cas est le système nerveux, avec la formation d'un ou de plusieurs abcès cérébraux, plus rarement de méningites. Lors des localisations multiples, la mortalité dépasse les 50%. Les autres sites de prédilection sont la peau, les tissus sous cutanés, les parties molles, les os et les articulations [8].

Nos 2 patients présentent une localisation secondaire sous cutanée avec abcès des parties molles, ceci a été rarement décrit chez les sujets immunocompétents. Cette forme reste l'apanage du sujet immunodéprimé par diffusion hématogène ou par contigüité suite au foyer pulmonaire dans 10 à 15% des cas [1]. Les manifestations radiologiques de nocardiose pulmonaire sont diversifiées et non spécifiques: un syndrome alvéolaire, des nodules, des cavitations, masses pulmonaires et un épanchement pleural [7]. La forme pseudotumorale retrouvée chez notre malade reste rare et cause ainsi une errance diagnostique d'autant plus que le malade est immunocompétent. Le diagnostic de nocardiose reste bactériologique, basé sur la microscopie et l´isolement de la culture, mais nocardia peut être confondue avec mycobacterium, principalement en raison de leur similitude à la fois clinique et bactériologique: la croissance sur milieu de Löwenstein-Jensen (LJ), la présence de bacilles acides à travers la coloration de Ziehl-Neelsen et la morphologie des colonies [9]. A l'examen direct de nocardia, les bacilles sont fins, ramifiés, gram positifs, le caractère d'acido-alcoolo-résistance des Nocardia est absent par la technique de Ziehl-Neelsen classique, mais partielle avec la technique modifiée de Kinyoun [1]. L'histologie reste un moyen diagnostic souvent sollicité comme c'est le cas de notre patient, souvent suite à la négativité des autres prélèvements. L'examen anatomopathologique retrouve des lésions pyogranulomateuses à centre abcédé, entourées de couronne de PNN et une deuxième couronne d'histiocytes dans un tissu fibreux, les filaments de nocardia sont mis en évidence par coloration de Gram et de Grocott [10]. Les Nocardia étant des bactéries ubiquitaires dans la nature, leur isolement en culture à partir d'expectorations ne reflète pas nécessairement une infection profonde et peut être la conséquence d'une colonisation respiratoire, voire d'une contamination de laboratoire. Dans une étude rétrospective menée dans un laboratoire de référence australien, aucune signification clinique n'a pu être relevée dans 20% des cas d'isolement de Nocardia spp [1]. Sur le plan thérapeutique, l'infection à nocardia nécessite une antibiothérapie systémique prolongée de 3 à 12 mois selon la localisation, la gravité et le terrain. L'association triméthoprim-sulfaméthoxazole constitue le traitement le plus anciennement prescrit vu la sensibilité de plusieurs espèces à ce traitement. Cependant, l'association classique amoxicillin-acide clavulanique plus amikacine, imipénème plus amikacine ou céfotaxime plus amikacine se sont révélées efficaces sur plusieurs souches, ensuite un relai par voie orale par triméthoprim-sulfaméthoxazole peut être proposé [1, 8].

## Conclusion

La nocardiose est une maladie rare, mais en évolution suite aux situations d'immunodépression. Elle pose jusqu'à présent un problème de retard diagnostic et de traitement qui reste long et mal codifié.

## Conflits d'intérêts

Les auteurs ne déclarent pas de conflits d'intérêt.
